# Real-Time Environment Monitoring Using a Lightweight Image Super-Resolution Network

**DOI:** 10.3390/ijerph18115890

**Published:** 2021-05-31

**Authors:** Qiang Yu, Feiqiang Liu, Long Xiao, Zitao Liu, Xiaomin Yang

**Affiliations:** 1 College of Electronics and Information Engineering, Sichuan University, Chengdu 610064, China; yqiang@stu.scu.edu.cn (Q.Y.); feiqiangliu@stu.scu.edu.cn (F.L.); 2 School of Aeronautics & Astronautics, Sichuan University, Chengdu 610064, China; 3 Science and Technology on Electronic Information Control Laboratory, Chengdu 610036, China; laizhibing@stu.scu.edu.cn; 4 TAL Education Group, Beijing 100080, China; liuzitao@100tal.com

**Keywords:** image super-resolution, real-time, deep learning, lightweight model, environment research, convolutional neural networks

## Abstract

Deep-learning (DL)-based methods are of growing importance in the field of single image super-resolution (SISR). The practical application of these DL-based models is a remaining problem due to the requirement of heavy computation and huge storage resources. The powerful feature maps of hidden layers in convolutional neural networks (CNN) help the model learn useful information. However, there exists redundancy among feature maps, which can be further exploited. To address these issues, this paper proposes a lightweight efficient feature generating network (EFGN) for SISR by constructing the efficient feature generating block (EFGB). Specifically, the EFGB can conduct plain operations on the original features to produce more feature maps with parameters slightly increasing. With the help of these extra feature maps, the network can extract more useful information from low resolution (LR) images to reconstruct the desired high resolution (HR) images. Experiments conducted on the benchmark datasets demonstrate that the proposed EFGN can outperform other deep-learning based methods in most cases and possess relatively lower model complexity. Additionally, the running time measurement indicates the feasibility of real-time monitoring.

## 1. Introduction

Environment research plays an important role in human daily activities. According to the results of environmental research, further analysis and effective solutions to existing problems can be made [1,2]. Image processing technology is widely used in environment research, such as remote sensing [3], object recognition [4] and classification [5]. All of these applications will performs better with higher resolution (HR) images since the HR images contain more information compared with low resolution (LR) images. However, the image directly obtained through the imaging devices is usually unsatisfactory due to the complex environment impacts and the limitation of sensors. Meanwhile, the high cost makes it hard to upgrade all the imaging devices. To alleviate the problem, an alternative method is image post-processing by super-resolution algorithms. Single image super-resolution (SISR) is an important research topic in the field of computer vision, whose goal is to restore a high resolution (HR) image from its low resolution (LR) counterparts. Essentially, SISR is a challenging ill-posed inverse problem that is hard to solve well. Despite the difficulty, there have been many attempts to tackle the SR problem since it has high value in the applications mentioned above.

With the resurgence of convolutional neural networks (CNNs) [5], deep learning (DL)-based methods have exhibited great advantages over traditional methods [6,7] in the image processing tasks. The first attempt to introduce CNN to SISR was made by Dong et al. [8]. Their method named SRCNN had a three-layer architecture. These three convolution layers completed the tasks of extracting features from LR image, non-linearly mapping the features to HR image space and reconstructing the HR image, respectively. Such a simple model had shown prominent results, which outperformed previous works by a large margin. Since then, DL has become the mainstream methods for SISR. One of the most intuitive ways to improve the performance is to deepen the networks, but deep neural network is usually hard to train and may suffer from the gradient vanishing/exploding problem.To address these issues, Kim et al. [9] proposed a very deep convolution network (VDSR) for SISR. This method provided an effective way to solve the problems encountered in building deep networks. Concretely, VDSR adopted a high learning rate and used gradient clipping technique to avoid gradient exploding. Besides, global residual learning was employed to ease the training burden. The success of VDSR indicates the usefulness of deep networks and residual learning. Based on the fundamental works [8,9], many deep SISR networks [10,11,12] have been proposed and achieved promising performance. Although deeper network contributes to better performance, it also leads to the large model size and heavy computational burden, hindering the application of SISR technology to mobile devices. One simple strategy to construct a lean network is to share model parameters in a recursive manner. Representative works of this method are deeply-recursive convolutional network for image super-resolution (DRCN) [13] and image super-resolution via deep recursive residual network (DRRN) [14]. They did show superior performance and compact the network model, but the computation was still large since there were many recursions. Thus, the design of lightweight CNN-based SISR networks should be portable and efficient.

**Figure 1:**
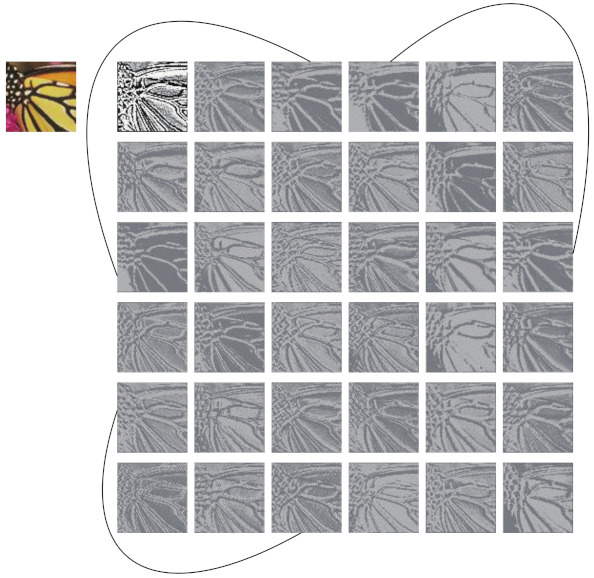
Visualization of some feature maps generated by the first residual block of RCAN. The feature map pairs connected by curves have strong similarity.

If one visualizes the feature maps of each convolutional layer of a well-trained CNN, it’s easy to find out that there is a certain relationship among the feature maps generated by the same convolutional layer, and this relationship is manifested by the similarity between some feature maps. As shown in Figure 1, we use the upper left image as the input of residual channel attention network for image super-resolution (RCAN) [11]. The following images are the feature maps generated by the first residual block of RCAN. It’s obviously that these feature maps contain the outline of the input image and most of them have similar contents. As we have annotated in Figure 1, the similar image pairs are connected with curves. One feature map in a pair can be approximately obtained from the other one through simple transformation [15]. It is these numerous and extremely similar feature maps that help the networks fully mine the information contained in the input image. However, the existing SISR methods have rarely taken the redundant feature maps into account.

In order to fill this gap, we design the efficient feature generating block (EFGB), which can leverage the intrinsic features to produce more feature maps in an economical way. Furthermore, to improve the performance of the model, we introduce the staged information refinement unit (SIRU) for better feature extraction. We establish the local residual module (LRM) based on the SIRU and the main body of proposed efficient feature generating network (EFGN) is constructed by stacking several LRMs. More details will be described in Section 3.

In summary, there are two main contributions of this work:

- The efficient feature generating block is proposed, which can generate more feature maps in an efficient way, so the network can achieve high performance while keeping low computation complexity.
- The Super Resolution Efficient Feature Generating Network is proposed, which introduces the staged information refinement unit to further boost the network performance.

The rest of the paper is organized as follows: Section 2 briefly reviews the works related to our method. Section 3 introduces the details of the proposed EFGN. Section 4 gives the experimental results and analysis. Finally, conclusions and future work are in Section 5.

## 2. Related Works

### 2.1. CNN-Based SISR Methods

Thanks to the rapid development of high-performance computing devices and the appearing of large-scale image datasets [16,17], training a deep convolutional neural network (CNN) for solving computer vision tasks becomes possible. DL-based methods have gained great advantages over the conventional methods in the field of computer vision. SISR is by no means an exception. Dong et al. [8] firstly proposed a deep convolutional network (SRCNN) for image SR and their method had achieved satisfactory results. VDSR [9] further improved the performance of SRCNN by deepening the network and introducing residual learning. DRCN [13] firstly introduced recursive learning to SISR for saving parameters. Later on, Tai et al. combined recursive learning and residual learning in DRRN [14] and outperformed the previous state-of-the-arts methods. All of above methods have one common drawback, the LR images is preprocessed to the size of HR images before being fed into the networks, which results in heavy computational burden and loss of information in original images. To tackle this issue, Dong et al. meliorated SRCNN by introducing a transposed convolution layer at the end of the network. This layer is also known as deconvolutional layer, which upsamples the feature maps to desired size in a learnable way, so the proposed accelerating the super-resolution convolutional neural network (FSRCNN) [18] was capable of taking the LR images as input. Almost at the same time, Shi et al. proposed real-time single image and video super-resolution using an efficient sub-pixel convolutional neural network (ESPCN) [19] for building a direct mapping from LR images to HR images. The efficiency was guaranteed by the newly designed sub-pixel layer, which could rearrange the feature maps at the end of the network to produce HR images. Thereafter, transposed convolution layer and sub-pixel layer have been basic components in many image SR networks. Another remarkable progress was made by enhanced deep residual networks for single image super-resolution (EDSR) [10], which put forward and confirmed the point that batch normalization (BN) is not suitable for SISR. Image super-resolution using dense skip connections (SRDenseNet) [20] introduced dense skip connections for reuse of feature maps. To further improve the performance of SR model, deeper networks with more sophisticated architectures were proposed. In residual dense network for image super-resolution (RDN) [12], hierarchical features were fully utilized with dense connections and multiple residual learning. Zhang et al. proposed RCAN [11], which incorporated channel attention mechanism and residual-in-residual structure to construct very deep networks and achieved outstanding results. When it comes to the case of lightweight networks, Hui et al. [21] proposed the information distillation network (IDN) for fast and accurate SISR. The main idea of IDN is splitting the feature maps into two parts, one of which is for further processing and the other is preserved. Ahn et al. [22] built a cascading residual network to pursue a trade-off between the efficiency and performance by using group convolution.

### 2.2. Efficient Convolutional Neural Network

With the emergency requirements of applying well-trained CNNs to embedded devices, lots of lightweight models have been proposed. In SqueezeNet [23], the dimension of feature maps is compressed by 1×1 convolution, so as to reduce the network parameters. The series of MobileNets [24] designed depth-wise separable convolutions by placing pointwise convolution after depthwise convolution for solving the problem of poor information flow. ShuffleNet [25] used channel shuffle operation to achieve the same effect as MobileNets. More recently, Han et al. proposed GhostNet [15] with introducing the novel ghost module, which could generate more features by using cheap operations. We have adopted this idea and improved it to make it more effective for SISR.

## 3. Methods

In this section, we give the overall network architecture and introduce the workflow of proposed method at first. Then we describe the local residual module in a top-down manner, which is the core part of our proposed method. At the beginning, we give a concise description of the symbols in Table 1.

### 3.1. Framework

**Figure 2:**
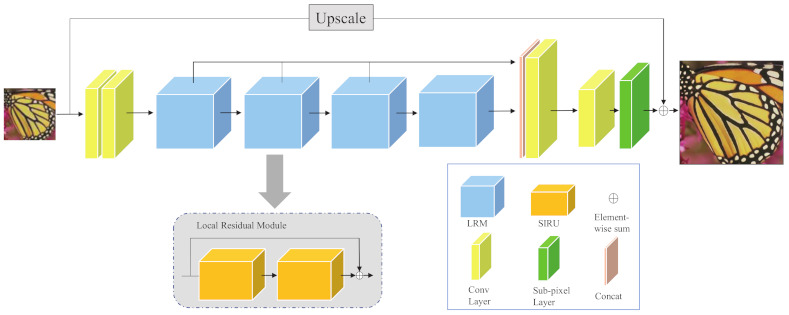
Network structure of proposed efficient feature generating network (EFGN).

As depicted in Figure 2, our EFGN is mainly composed of three parts: a primary feature extraction module (FEM), several stacked local residual modules (LRMs) and a reconstruction module (RM). Given an input image $I_{LR}$, two convolution layers with kernel size of 3×3 are utilized to extract primary features from the input image, which can be expressed by

(1) $$H_{0}=F_{E}\left(I_{LR}\right)=Conv\left(Conv\left(I_{LR}\right)\right),$$

$F_{E}\left(\cdot \right)$ is the function of FEM. This operation essentially increases the channel dimension of $I_{LR}$ and then the extracted feature $H_{0}$ is used for further processing with LRMs. This procedure can be expressed by the following equation:

(2) $$H_{k}=F_{R}^{k}\left(H_{k-1}\right),\;k=1,2,\ldots ,K,$$

where $F_{R}^{k}\left(\cdot \right)$ denotes the function of *k*-th LRM, $H_{k-1}$ and $H_{k}$ are the input and output of *k*-th LRM respectively. In the RM, all outputs from previous LRMs are collected by the feature gathering block with concatenation operation, then a 1×1 convolution layer is used to fuse the aggregated features. The process can be expressed by the following mathematical formula:

(3) $$H_{L}=F_{1\times 1}\left([H_{1},H_{2},\ldots ,H_{k}]\right),\;k=1,2,\ldots ,K,$$

where [·] represents the concatenation operation in channel-wise, which keeps all the information from previous modules without any loss. As a result, the later 1×1 convolution layer can make full use of the hierarchical features contained in preceding LRMs. This scheme bring about the benefit of performance boosting with parameters slightly increasing. At last, a convolution layer followed by a sub-pixel convolution layer are taken as the reconstruction block to reconstruct the fused features $H_{L}$. Hence, the SR image can be obtained by

(4) $$I_{SR}=F_{up}\left(H_{L}\right)+B\left(I_{LR}\right)=F_{EFGN}\left(I_{LR}\right),$$

where $F_{up}\left(\cdot \right)$ and B(·) denote the reconstruction block and bicubic interpolation operation respectively. $F_{EFGN}\left(\cdot \right)$ is the function of proposed EFGN.

Previous studies [10,12,21,22,26] have proven that L1 (MAE) loss is more suitable for SR task than L2 (MSE) loss, since L1 loss leads to better convergence and more satisfactory results. We follow their steps and employ the L1 loss as the loss function during the training process. Given a training set ${\left\{I_{LR}^{j},I_{HR}^{j}\right\}}_{j=1}^{N}$ of N pairs LR-HR images. Then, in a certain training epoch, the loss function can be expressed as follows:

(5) $$L\left(\Theta \right)=\frac{1}{N}\sum_{j=1}^{N}{∥F_{EFGN}\left(I_{LR}^{j}\right)-I_{HR}^{j}∥}_{1}$$

where Θ is the network parameters to be optimized.

### 3.2. Local Residual Module

As shown in Figure 2, the main component of the proposed EFGN, local residual module (LRM), is constructed by two staged information refinement unit (SIRU). Skip connection is adopted in the module to make the residual branch focus on distinguishing features. We then give more details about SIRU and its inner structure, i.e., the efficient feature generating block (EFGB).

#### 3.2.1. Staged Information Refinement Unit

**Figure 3:**
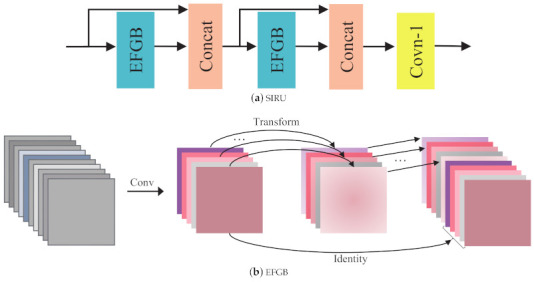
(**a**) The architecture of SIRU. (**b**) An illustration of efficient feature generating block.

In deep CNN, feature maps from relative shallow layers usually contain abundant texture information. It’s vital to use these informative feature maps when we are processing reconstruction tasks. Moreover, previous works [20,27] have verified that the reuse of features is helpful for building compact models. In order to efficiently utilize the feature maps from previous layers, we exploit a stage-like structure, in which feature maps from previous step will guide the reconstruction of later steps. Its graphic depiction is shown in Figure 3a. Denoting the input of *k*-th LRM as $H_{k,0}$, the output of first SIRU in this LRM can be gotten by

(6) $$\begin{array}{cc} \; & H_{k,1}^{stage\_1}=\left[B_{k,1}^{stage\_1}\left(H_{k,0}\right),H_{k,0}\right],\\ \; & H_{k,1}^{stage\_2}=\left[B_{k,1}^{stage\_2}\left(H_{k,1}^{stage\_1}\right),H_{k,1}^{stage\_1}\right],\\ \; & H_{k,1}=f_{k,1}\left(H_{k,1}^{stage\_2}\right), \end{array}$$

where $B_{k,1}^{stage\_s}\left(\cdot \right)$ indicates the *s*-th EFGB of first SIRU in the *k*-th LRM, the $f_{k,1}\left(\cdot \right)$ denotes the 1×1 convolution layer used for compression. $H_{k,1}^{stage\_s}$ represents the refined information of *s*-th stage, which is the concatenation along channel dimension of the input and output of $B_{k,1}^{stage\_s}\left(\cdot \right)$, and the $H_{k,1}$ is the output of SIRU. Similarly, we can get the $H_{k,2}$. The final output of *k*-th LRM can be obtained by

(7) $$H_{k}=H_{k,0}+H_{k,2}=F_{R}^{k}\left(H_{k,0}\right),$$

where $F_{R}^{k}\left(\cdot \right)$ is the function of *k*-th LRM. As shown in Figure 3, EFGB denotes the efficient feature generating block. Concat means concatenation operation. Conv-1 is the 1×1 convolution layer. It forms a stage-like architecture. Features from different levels that contain collective information would boost the image SR performance.

#### 3.2.2. Efficient Feature Generating Block

The mainstream SR methods using conventional convolution would produce redundant feature maps as shown in Figure 1, which will take up a lot of resources. We tend to generate these redundancy in a more efficient way. We use a similar approach to Ghostnet [15], but two modifications are made. (i) The batch normalization layers are removed. (ii) We replace the 1×1 convolution layer with 3×3 convolution layer to obtain intrinsic feature maps, so as to increase the size of receptive field, which have proven to be important for image SR in [9]. As illustrated in Figure 3b, the input feature maps are $X\in R^{c\times w\times h}$, where *c* denotes the number of channels, *w* and *h* are the width and height of *X* respectively. An ordinary convolution layer is firstly applied on the input data to yield the intrinsic feature maps $M\in R^{\frac{c}{2}\times w\times h}$ (note that proper padding is set to retain the spatial size of feature maps), this process can be formulated as

(8) M=f(X),

where $f\in R^{c\times k\times k\times \frac{c}{2}}$ denotes the convolution filter with kernel size k×k, bias term is omitted for clarity. Then each feature map of *M* is transformed to its potential counterpart. In practice, the transformation is implemented by depthwise convolution (DWC). The DWC is a special case of group convolution, which process each feature map separately. In other words, the group of DWC is equal to the number of input channels. Finally, the output *Y* of EFGB is consist of the identity mapping of *M* and its variant, it can be expressed as

(9) Y=[D(M),M],

where $D\in R^{\frac{c}{2}\times k\times k}$ denote the function of depthwise convolution.

## 4. Experiments

### 4.1. Datasets and Metrics

Since the DIV2K [16] dataset was proposed, it has been widely used to train SISR networks due to its diversity and high quality. As most recent works [10,12,22] do, the 800 training images from DIV2K datasets are chosen as training set in our experiments. We test the trained models on four standard benchmarks: Set5 [28], Set14 [29], B100 [30] and Urban100 [31]. For a fair comparison with other methods, we use the peak signal-to-noise ratio (PSNR) and the structural similarity index (SSIM) [32] as evaluation metrics. Because human eye is more sensitive to luminance information, the SR results are converted to YCbCr space at first. Then the PSNR and SSIM are computed on the luminance channel (i.e., the Y channel of YCbCr space).

### 4.2. Implementation Details

The detailed structure of LRM is described in Section 3.2. We now give the parameter settings for the rest of the network. Since we intend to process RGB images, the first convolution layer of FEM has a size of $3\times k\times k\times n_{f}$. Where *k* is the kernel size of convolution filter and $n_{f}$ is the number of output channels. In this paper, all convolution kernel sizes are 3×3 except for the specified 1×1 convolution layers. For the following one convolution layer and next *T* LRMs, the input and output both are feature maps with channel number $n_{f}$. We set $n_{f}$ as 64 to achieve a trade-off between the model size and performance. The 1×1 convolution layer in feature gathering block compress the $T\cdot n_{f}$ feature maps to $n_{f}$. In the upsample block, different scale factors adopt different settings. If the scale factor is r, a convolution layer is first adopted to produce feature maps with the channel number of $c\cdot r^{2}$, c is the channel of output images. In the case of output RGB images, c is equal to 3. Then the sub-pixel layer will periodically shuffle the feature maps to reconstruct the upscaled residual images. ReLU [33] function is employed as the activation function following all of the convolution layers except the convolution layer in upsample block.

The LR training images are downsampled from their HR counterparts with bicubic interpolation. We crop the image patches with a size of 48×48 from LR training images as the input. In each training iteration, 16 LR image patches are sent to the network. To further improve the generalization ability of our models, we carry out data argumentation on the image patches with random horizontal flips and $90^{\circ }$ rotations. The Adam optimizer [34] is applied to train our networks, the hyper-parameters $\beta_{1}$ and $\beta_{2}$ are set as 0.9 and 0.999 respectively. We use $2\times 10^{-4}$ as the initial learning rate and halve it every $2\times 10^{5}$ training iterations. The total training iterations are $8\times 10^{5}$. The whole training process are implemented on an NVIDIA 2080Ti GPU by Pytorch framework. The code is available at https://github.com/gokuson77/EFGN (accessed on 17 May 2021).

### 4.3. Efficiency Analysis

For a more intuitive comparison, we calculate the parameters and computational cost of a conventional convolution layer and our proposed EFGB. Assuming both of the input and output data have a shape of c×w×h, all convolution filters possess the kernel size of k×k. The trainable parameters of conventional convolution is $P_{C}=k^{2}\cdot c^{2}$, while the computational cost is $F_{C}=k^{2}\cdot c^{2}\cdot h\cdot w$. According to the above definition, the parameters of EFGB can be calculated by $P_{E}=k^{2}\cdot \frac{c^{2}}{2}+k^{2}\cdot \frac{c}{2}$, the corresponding computational cost is $F_{E}=k^{2}\cdot \frac{c^{2}}{2}\cdot h\cdot w+k^{2}\cdot \frac{c}{2}\cdot h\cdot w$. Then we can figure out the compression ratio $r_{c}$ and speed-up ratio $r_{s}$,

(10) $$\begin{array}{cc} \; & r_{c}=P_{C}/P_{E}=\frac{k^{2}\cdot c^{2}}{k^{2}\cdot \frac{c^{2}}{2}+k^{2}\cdot \frac{c}{2}}=\frac{2c}{c+1},\\ \; & r_{s}=F_{C}/F_{E}=\frac{k^{2}\cdot c^{2}\cdot h\cdot w}{k^{2}\cdot \frac{c^{2}}{2}\cdot h\cdot w+k^{2}\cdot \frac{c}{2}\cdot h\cdot w}=\frac{2c}{c+1} \end{array}$$

since *c* denotes the number of channels and c≫1, we can get $r_{c}\approx r_{s}\approx 2$, which means the proposed EFGB can roughly reduce the computation and model size by two times.

**Figure 4:**
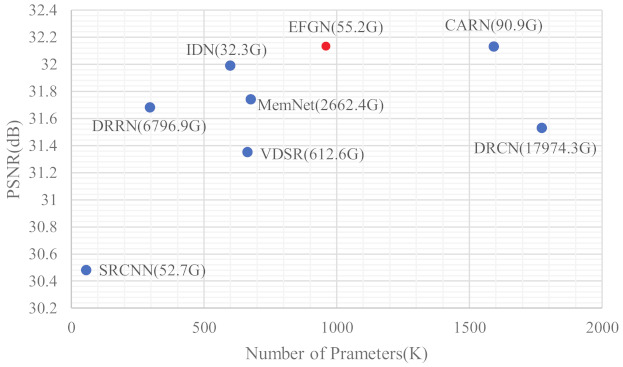
Trade-off between the model complexity and reconstruction performance with scale factor ×4 on Set5. The number after each method indicates the computational costs.

**Figure 5:**
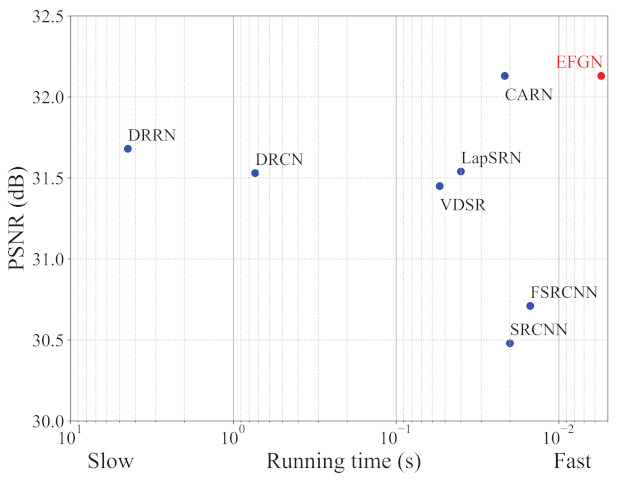
The trade-off between the running time and PSNR with scale factor ×4 on Set5.

Figure 4 shows the relationship of model complexity and reconstruction performance, from which we can observe that the EFGN can achieve a trade-off between the model complexity and reconstruction performance. It should be declared that we represent the computational costs by multiply-accumulate operations (MAC) and when calculating MAC the SR image is assumed to be 720P (1280×720). Furthermore, we also test the running time of some typical CNN-based SR methods. To be fair, all of the methods are tested on an NVIDIA 1080Ti GPU. The results are shown in Figure 5. It’s obviously that our proposed EFGN has the fastest running time (0.0055 s) while keeping high performance, which indicate the theoretical feasibility of applying the EFGN to real time monitoring.

### 4.4. Study of LRM

As the core of our proposed network, the LRM is worthy to be comprehensively investigated. Specifically, the parameter setting of SIRU in each LRM and the number of LRM (denote as T, e.g., T3 represents the model has three LRMs) in the network are discussed.

To find a proper parameter setting, we fix the input channel of first EFGB in SIRU as 64 and T as 4. Then we divide the model to three types according to the output of EFGB in SIRU. Concretely, the output channel of EFGB in expansion model is increased by 32. On the contrary, the reduction model reduces the output channel of EFGB by 32 while the basic model maintains the number of output channel as the same of input channel. The experiment results are presented in Table 2. EFGN_B is the basic model. EFGN_S and EFGN_L are the reduction model and expansion model, respectively. We can see that model with larger output channel numbers shows better performance. Meanwhile, the model size has also increased. The improvement between the EFGN_S and EFGN_B is significant (PSNR: +0.13 dB, Parameters: +496 K). In contrast, the performance improvement brought by parameter increase between EFGN_B and EFGN_L (PSNR: +0.05 dB, Parameters: +643 K) is less cost-effective. So, the setting of EFGN_B is chosen for further experiments.

**Figure 6:**
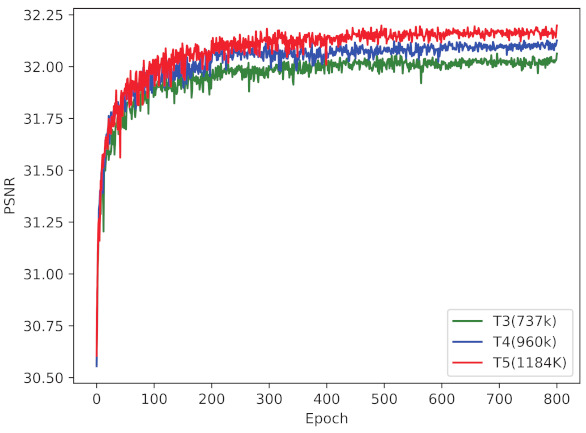
Converge analysis of EFGN with different number of LRM. The parameter of each model is also listed. The results are evaluated on Set5 dataset with ×4 scale.

We then investigate the impact of the number of LRM. As illustrated in Figure 6, there are distinguished gaps between different models, which indicates deeper networks achieve better performance. For a fair comparison with other models, we select the model with 4 LRMs in subsequent experiments.

### 4.5. Effects of SIRU and EFGB

To verify the effectiveness of proposed staged information refinement unit (SIRU) and efficient feature generating block (EFGB), we construct two networks for ablation study. It is worth noting that both of the ablation models follow the same training settings as the EFGN to ensure the credibility of experimental results.

**Figure 7:**
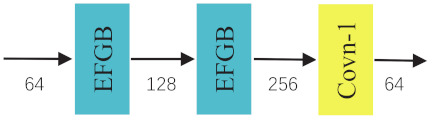
The structure of modified SIRU. 64, 128, 256 represent the channel numbers of feature maps.

The first one called EFGN_NS has the modified SIRU as illustrated in Figure 7. It is easy to find out that the concatenation operations are removed compared with the original one. As a result, the shallow features can not directly propagate to the deeper layers, leading to loss of the staged feature reuse mechanism. Worse still, to maintain the number of input and output channels in every layers needs more parameters. From Table 3, we can observe that the network with SIRU structure achieves better performance and consumes less parameters (Note that the number of LRM in EFGN_NS are three.).

**Figure 8:**
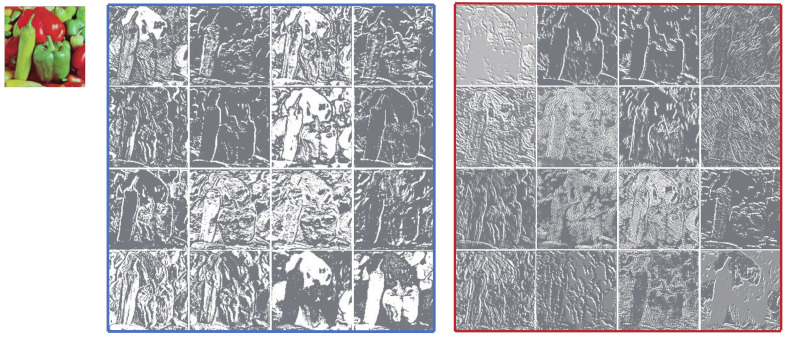
Visualization of the feature map generated by the first EFGB in the first LRM.

The other model named EFGN_NE uses ordinary convolution layers for features extraction instead of the EFGB. As we have analyzed in Section 3.2.2, this change will also bring about the network parameters increment. So for a fair comparison, the EFGN_NE adjusts the number of SIRU in each LRM to one. The results are recorded in Table 3. Compared with EFGN_NE, the EFGN only increases a few parameters but in exchange for prominent performance improvement. In detail, the network structure of EFGN is deeper than EFGN_NE. But thanks to the EFGB, we can compact the model to find a trade-off between performance and efficiency. Figure 8 shows the intrinsic and transformed feature maps in the EFGB. The left top image is the input. The feature maps in blue box are the intrinsic features. The feature maps in the red box are the transformed features. We can find that the intrinsic features mainly focus on low frequency information. The transformed feature maps contain both high and low frequency information, which proves the EFGB is qualified to extract features from input image.

### 4.6. Quantitative and Qualitative Evaluation

We compare our method with several DL-based SR algorithms. The quantitative comparison is evaluated by metrics mentioned above (PSNR and SSIM). For a comprehensive study, we also investigate the network parameters and computational costs (the calculation of computational costs is the same as stated in Section 4.3). The detailed results are shown in Table 4. As we can see, the methods with recursive mechanism, such as DRRN, MemNet, DRCN, has extremely large computations but their performance is not very satisfactory. Compared with the lightweight models, our proposed EFGN has the best performance on most benchmark datasets for all scaling factors. Meanwhile, the model complexity of EFGN is at the medium level, which demonstrates the efficiency of proposed method.

**Figure 9:**
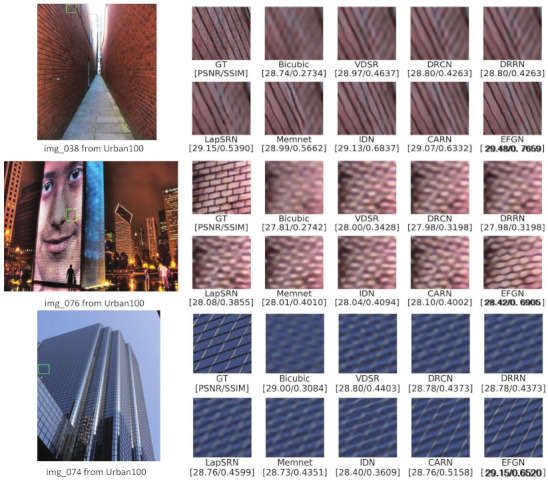
Qualitative comparison of EFGN with other deep learning-based methods on ×4 SISR.

The qualitative evaluation is carried out by comparing the reconstructed SR images visually. Some results are shown in Figure 9. In “img_038”, the image obtained by our proposed EFGN is close to the GT images but the other methods have failed to recover the sharp lines. In the results of “img_076”, all the previous methods reconstruct the details of image in wrong direction while our method recover the clear and correct textures. For the result of “img_074”, the images obtained by other methods are suffering from distortion and blurriness while our EFGN can recover a more faithful image. That further indicates the effectiveness of EFGN.

### 4.7. Evaluation of Object Recognition

An effective method of environmental monitoring is the recognition of objects in the environment. Based on the result of object recognition, proper actions can be taken. The image SR algorithms can pre-process images for object recognition to achieve higher accuracy. We use different SR algorithms to process the images used for recognition and evaluate the performance of object recognition models to indicate the practicability of our proposed method in environmental monitoring.

**Figure 10:**
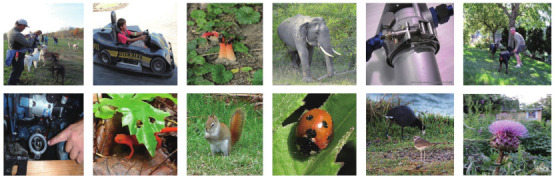
Images from ImageNet CLS-LOC validation dataset.

**Figure 11:**
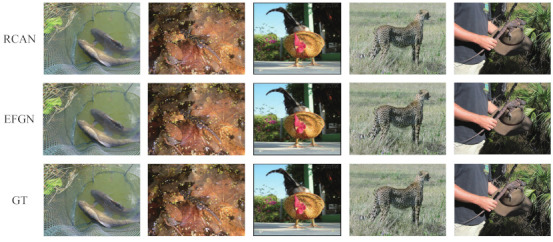
Visual comparison of RCAN with our proposed EFGN at ×4 super-resolution on images from ImageNet.

For the comparison, we use the ResNet-50 (The pretrained model of ResNet-50 is released by Pytorch) [4] as the object recognition model. The test images are chosen from the ImageNet CLS-LOC validation dataset. The dataset has 50,000 images and we only use the first 1000 images for testing. Some of the images are listed in Figure 10. Each image has one exact label in 1000 categories. Firstly, we downscale these images with a scaling factor of 4. Then the downscaled images are reconstructed by different SR algorithms. Finally, the reconstructed images are fed into the ResNet-50 for recognition. The results are shown in Table 5. The lower Top-1(5) error indicates better results. As expected, the bicubic interpolation method has the worst result. But surprisingly, our proposed method outperforms RCAN (The results are produced by RCAN official code) [11] which yields images with relatively high PSNR score. We have made further analysis to find the reason. As shown in Figure 11, the images reconstructed by RCAN in first row are over smooth that contain annoying artifacts. Meanwhile, the images generated by EFGN in second row are closer to the ground truth (GT) images. So higher object recognition score can be obtained. The results demonstrate our proposed method is able to be used for environment monitoring.

## 5. Conclusions and Future Work

In this paper, we propose a lightweight image super-resolution network for real-time environment monitoring. Concretely, a novel efficient feature generating block is designed to fully utilize the redundancy among feature maps of the same layer. Additionally, the staged information refinement unit is introduced to explore hierarchical information reuse, which can further boost the reconstruction performance. Extensive experiments conducted on the benchmarks demonstrate that the proposed EFGN surpasses other DL-based methods while balancing the SR reconstruction performance with network parameters and computational costs. Meanwhile, our method is capable of real-time monitoring since it has fast running speed. Although the model shows excellent performance, a room of it to improve still exists. First and foremost, we only use L1 loss to train our network, which cannot restore the high frequency details in some cases. In order to produce more visually friendly images, we plan to incorporate generative adversarial network (GAN) and use joint loss to train our network. Besides, the gradient information in LR images have been proven to be helpful in the image SR task. In the future, we will make an attempt to exploit the informative gradient features for better reconstruction.

## Figures and Tables

**Table 1:** Concise description of symbols in the paper.

Symbols	Description
$H_{0}$	The extracted primary features
$I_{LR}$	LR images
$F_{E}\left(\cdot \right)$	Function of FEM
Conv	Convolution layer
$F_{R}^{k}\left(\cdot \right)$	Function of *k*-th LRM
$H_{k}$	Output of *k*-th LRM
[·]	Concatenation operation in channel-wise
$F_{1\times 1}\left(\cdot \right)$	1×1 convolution layer
$H_{L}$	Fused features
$F_{up}\left(\cdot \right)$	Function of reconstruction block
B(·)	Bicubic interpolation
$F_{EFGN}\left(\cdot \right)$	Function of proposed EFGN
$I_{SR}$	SR image
$I_{HR}$	HR image
$H_{k,0}$	Input of *k*-th LRM
$B_{k,1}^{stage\_s}\left(\cdot \right)$	*s*-th EFGB of first SIRU in the *k*-th LRM
$f_{k,1}\left(\cdot \right)$	1×1 convolution layer in *k*-th LRM
$H_{k,1}^{stage\_s}$	Refined features of *s*-th EFGB of first SIRU in the *k*-th LRM

**Table 2:** Results of different parameter setting in SIRU on Set5 dataset with scaling factor 4.

	EFGN_S	EFGN_B	EFGN_L
Parameters	464 K	960 K	1603 K
PSNR	32.00	32.13	32.18

**Table 3:** Investigations of SIRU scheme and EFGB module for scale factor ×4 on Set5 dataset. w/ and w/o represent with and without, respectively.

Model	SIRU	EFGB	Parameters	PSNR
EFGN_NS	w/o	w/	1303 K	32.08
EFGN_NE	w/	w/o	887 K	31.98
EFGN	w/	w/	960 K	32.13

**Table 4:** Quantitative results for scale factor ×2, ×3 and ×4 on benchmarks. Best and second best results are **bold** and underlined.

Method	Scale	Params	MAC	Set5 PSNR/SSIM	Set14 PSNR/SSIM	B100 PSNR/SSIM	Urban100 PSNR/SSIM
Bicubic	2	-	-	33.65/0.9299	30.34/0.8688	29.56/0.8431	26.88/0.8403
SRCNN [8]	2	57 K	52.7 G	36.66/0.9542	32.45/0.9067	31.36/0.8879	29.50/0.8946
FSRCNN [18]	2	12 K	6.0 G	37.00/0.9558	32.63/0.9088	31.53/0.8920	29.88/0.9020
VDSR [9]	2	665 K	612.6 G	37.53/0.9587	33.03/0.9124	31.90/0.8960	30.76/0.9140
DRCN [13]	2	1774 K	17,974.3 G	37.63/0.9588	33.04/0.9118	31.85/0.8942	30.75/0.9133
LapSRN [35]	2	813 K	29.9 G	37.52/0.9591	32.99/0.9124	31.80/0.8952	30.41/0.9103
DRRN [14]	2	297 K	6796.9 G	37.74/0.9591	33.23/0.9136	32.05/0.8973	31.23/0.9188
MemNet [36]	2	677 K	2662.4 G	37.78/0.9597	33.28/0.9142	32.08/0.8978	31.31/0.9195
IDN ^1^ [21]	2	579 K	124.6 G	37.85/0.9598	33.58/**0.9178**	32.11/0.8989	31.95/0.9266
CARN [22]	2	1592 K	222.8 G	37.76/0.9590	33.52/0.9166	32.09/0.8978	31.92/0.9256
EFGN(Ours)	2	939 K	216 G	**38.01**/**0.9604**	**33.59**/0.9172	**32.17**/**0.8995**	**32.03**/**0.9275**
Bicubic	3	-	-	30.39/0.8682	27.55/0.7742	27.21/0.7385	24.46/0.7349
SRCNN [8]	3	57 K	52.7 G	32.75/0.9090	29.30/0.8215	28.41/0.7863	26.24/0.7989
FSRCNN [18]	3	12 K	5.0 G	33.18/0.9140	29.37/0.8240	28.53/0.7910	26.43/0.8080
VDSR [9]	3	665 K	612.6 G	33.66/0.9213	29.77/0.8314	28.82/ 0.7976	27.14/0.8279
DRCN [13]	3	1774 K	17,974.3 G	33.82/0.9226	29.76/0.8311	28.80/0.7963	27.15/0.8276
DRRN [14]	3	297 K	6796.9 G	34.03/0.9244	29.96/0.8349	28.95/0.8004	27.53/0.8378
MemNet [36]	3	677 K	2662.4 G	34.09/0.9248	30.00/0.8350	28.96/0.8001	27.56/0.8376
IDN ^1^ [21]	3	588 K	56.3 G	34.24/0.9260	30.27/0.8408	29.03/0.8038	27.99/0.8489
CARN [22]	3	1592 K	118.8 G	34.29/0.9255	**30.29**/0.8407	29.06/0.8034	27.38/0.8404
EFGN(Ours)	3	948 K	96.7 G	**34.36**/**0.9268**	30.28/**0.8411**	**29.08**/**0.8048**	**28.10**/**0.8514**
Bicubic	4	-	-	28.42/0.8104	26.00/0.7027	25.96/0.6675	23.14/0.6577
SRCNN [8]	4	57 K	52.7 G	30.48/0.8628	27.50/0.7513	26.90/0.7101	24.52/0.7221
FSRCNN [18]	4	12 K	4.6 G	30.71/0.8657	27.59/0.7535	26.98/0.7150	24.62/0.7280
VDSR [9]	4	665 K	612.6 G	31.35/0.8838	28.01/0.7674	27.29/0.7251	25.18/0.7524
DRCN [13]	4	1774 K	17,974.3 G	31.53/0.8854	28.02/0.7670	27.23/0.7233	25.14/0.7510
LapSRN [35]	4	813 K	149.4 G	31.54/0.8852	28.09/0.7700	27.32/0.7275	25.21/0.7562
DRRN [14]	4	297 K	6796.9 G	31.68/0.8888	28.21/0.7720	27.38/0.7284	25.44/0.7638
MemNet [36]	4	677 K	2662.4 G	31.74/0.8893	28.26/0.7723	27.40/0.7281	25.50/0.7630
IDN ^1^ [21]	4	600 K	32.3 G	31.99/0.8928	28.52/0.7794	27.52/0.7339	25.92/0.7801
CARN [22]	4	1592 K	90.9 G	32.13/0.8937	**28.60**/0.7806	**27.58**/0.7349	**26.07**/0.7837
EFGN(Ours)	4	960 K	55.2 G	**32.13**/**0.8945**	28.57/**0.7810**	27.57/**0.7357**	26.03/**0.7846**

^1^ IDN refers to the results given by LatticeNet [37].

**Table 5:** ResNet-50 object recognition performance. The original images are served as baseline. Best results are shown in bold.

Metric	Bicubic	RCAN [11]	EFGN	Baseline
Top-1 error	0.366	0.344	**0.304**	0.238
Top-5 error	0.143	0.136	**0.098**	0.066

## Data Availability

Not applicable.

## References

[1] Xiong J., Hswen Y., Naslund J.A. (2020). Digital Surveillance for Monitoring Environmental Health Threats: A Case Study Capturing Public Opinion from Twitter about the 2019 Chennai Water Crisis. Int. J. Environ. Res. Public Health.

[2] Liu F., Yu Q., Chen L., Jeon G., Albertini M.K., Yang X. (2021). Aerial image super-resolution based on deep recursive dense network for disaster area surveillance. Pers. Ubiquitous Comput..

[3] Lai Z., Chen L., Jeon G., Liu Z., Zhong R., Yang X. (2021). Real-time and effective pan-sharpening for remote sensing using multi-scale fusion network. J. Real-Time Image Process..

[4] He K., Zhang X., Ren S., Sun J. Deep residual learning for image recognition. Proceedings of the IEEE Conference on Computer Vision and Pattern Recognition.

[5] Krizhevsky A., Sutskever I., Hinton G.E. (2012). Imagenet classification with deep convolutional neural networks. Adv. Neural Inf. Process. Syst..

[6] Wu J., Anisetti M., Wu W., Damiani E., Jeon G. (2016). Bayer demosaicking with polynomial interpolation. IEEE Trans. Image Process..

[7] Wang J., Wu J., Wu Z., Anisetti M., Jeon G. (2018). Bayesian method application for color demosaicking. Opt. Eng..

[8] Dong C., Loy C.C., He K., Tang X. (2014). Learning a deep convolutional network for image super-resolution. European Conference on Computer Vision.

[9] Kim J., Kwon Lee J., Mu Lee K. Accurate image super-resolution using very deep convolutional networks. Proceedings of the IEEE Conference on Computer Vision and Pattern Recognition.

[10] Lim B., Son S., Kim H., Nah S., Mu Lee K. Enhanced deep residual networks for single image super-resolution. Proceedings of the IEEE Conference on Computer Vision and Pattern Recognition Workshops.

[11] Zhang Y., Li K., Li K., Wang L., Zhong B., Fu Y. Image super-resolution using very deep residual channel attention networks. Proceedings of the European Conference on Computer Vision (ECCV).

[12] Zhang Y., Tian Y., Kong Y., Zhong B., Fu Y. Residual dense network for image super-resolution. Proceedings of the IEEE Conference on Computer Vision and Pattern Recognition, Salt Lake City.

[13] Kim J., Kwon Lee J., Mu Lee K. Deeply-recursive convolutional network for image super-resolution. Proceedings of the IEEE Conference on Computer Vision and Pattern Recognition.

[14] Tai Y., Yang J., Liu X. Image super-resolution via deep recursive residual network. Proceedings of the IEEE Conference on Computer Vision and Pattern Recognition Workshops.

[15] Han K., Wang Y., Tian Q., Guo J., Xu C., Xu C. GhostNet: More Features from Cheap Operations. Proceedings of the 2020 IEEE/CVF Conference on Computer Vision and Pattern Recognition (CVPR).

[16] Agustsson E., Timofte R. NTIRE 2017 Challenge on Single Image Super-Resolution: Dataset and Study. Proceedings of the IEEE Conference on Computer Vision and Pattern Recognition Workshops.

[17] Deng J., Dong W., Socher R., Li L., Kai L., Li F. ImageNet: A large-scale hierarchical image database. Proceedings of the 2009 IEEE Conference on Computer Vision and Pattern Recognition.

[18] Dong C., Loy C.C., Tang X. (2016). Accelerating the super-resolution convolutional neural network. European Conference on Computer Vision.

[19] Shi W., Caballero J., Huszár F., Totz J., Aitken A.P., Bishop R., Rueckert D., Wang Z. Real-time single image and video super-resolution using an efficient sub-pixel convolutional neural network. Proceedings of the IEEE Conference on Computer Vision and Pattern Recognition.

[20] Tong T., Li G., Liu X., Gao Q. Image super-resolution using dense skip connections. Proceedings of the IEEE Conference on Computer Vision and Pattern Recognition Workshops.

[21] Hui Z., Wang X., Gao X. Fast and accurate single image super-resolution via information distillation network. Proceedings of the IEEE Conference on Computer Vision and Pattern Recognition, Salt Lake City.

[22] Ahn N., Kang B., Sohn K.A. Fast, accurate, and lightweight super-resolution with cascading residual network. Proceedings of the European Conference on Computer Vision (ECCV).

[23] Iandola F.N., Han S., Moskewicz M.W., Ashraf K., Dally W.J., Keutzer K. (2016). SqueezeNet: AlexNet-level accuracy with 50x fewer parameters and <0.5 MB model size. arXiv.

[24] Howard A.G., Zhu M., Chen B., Kalenichenko D., Wang W., Weyand T., Andreetto M., Adam H. (2017). Mobilenets: Efficient convolutional neural networks for mobile vision applications. arXiv.

[25] Zhang X., Zhou X., Lin M., Sun J. Shufflenet: An extremely efficient convolutional neural network for mobile devices. Proceedings of the IEEE Conference on Computer Vision and Pattern Recognition, Salt Lake City.

[26] Zhao H., Gallo O., Frosio I., Kautz J. (2016). Loss functions for image restoration with neural networks. IEEE Trans. Comput. Imaging.

[27] Huang G., Liu Z., Van Der Maaten L., Weinberger K.Q. Densely connected convolutional networks. Proceedings of the IEEE Conference on Computer Vision and Pattern Recognition Workshops.

[28] Bevilacqua M., Roumy A., Guillemot C., Morel M.L.A. Low-Complexity Single-Image Super-Resolution based on Nonnegative Neighbor Embedding. Proceedings of the British Machine Vision Conference (BMVC).

[29] Zeyde R., Elad M., Protter M. (2010). On single image scale-up using sparse-representations. International Conference on Curves and Surfaces.

[30] Martin D., Fowlkes C., Tal D., Malik J. A database of human segmented natural images and its application to evaluating segmentation algorithms and measuring ecological statistics. Proceedings of the Eighth IEEE International Conference on Computer Vision. ICCV 2001.

[31] Huang J.B., Singh A., Ahuja N. Single image super-resolution from transformed self-exemplars. Proceedings of the IEEE Conference on Computer Vision and Pattern Recognition.

[32] Wang Z., Bovik A.C., Sheikh H.R., Simoncelli E.P. (2004). Image quality assessment: From error visibility to structural similarity. IEEE Trans. Image Process..

[33] Nair V., Hinton G.E. Rectified linear units improve restricted boltzmann machines. Proceedings of the 27th International Conference on Machine Learning.

[34] Kingma D.P., Ba J. (2014). Adam: A method for stochastic optimization. arXiv.

[35] Lai W.S., Huang J.B., Ahuja N., Yang M.H. Deep laplacian pyramid networks for fast and accurate super-resolution. Proceedings of the IEEE Conference on Computer Vision and Pattern Recognition Workshops.

[36] Tai Y., Yang J., Liu X., Xu C. Memnet: A persistent memory network for image restoration. Proceedings of the IEEE Conference on Computer Vision and Pattern Recognition Workshops.

[37] Luo X., Xie Y., Zhang Y., Qu Y., Li C., Fu Y., Vedaldi A., Bischof H., Brox T., Frahm J.M. (2020). LatticeNet: Towards Lightweight Image Super-Resolution with Lattice Block. Computer Vision—ECCV 2020.

